# Microbial synthesis of poly-γ-glutamic acid (γ-PGA) with fulvic acid powder, the waste from yeast molasses fermentation

**DOI:** 10.1186/s13068-020-01818-5

**Published:** 2020-10-28

**Authors:** Yazhou Li, Jianghan Wang, Na Liu, Luxin Ke, Xiuyun Zhao, Gaofu Qi

**Affiliations:** 1 College of Life Science and Technology, Huazhong Agricultural University, Wuhan, 430070 China; 2 Department of Genetics and Genome Sciences, School of Medicine, Case Western Reserve University, Cleveland, OH 44106 USA

**Keywords:** Molasses, Fulvic acid powder, Poly-γ-glutamic acid (γ-PGA), *Bacillus velezensis*, Induced systemic resistance (ISR), Ethylene (ET) signaling

## Abstract

**Background:**

Molasses is a wildly used feedstock for fermentation, but it also poses a severe wastewater-disposal problem worldwide. Recently, the wastewater from yeast molasses fermentation is being processed into fulvic acid (FA) powder as a fertilizer for crops, but it consequently induces a problem of soil acidification after being directly applied into soil. In this study, the low-cost FA powder was bioconverted into a value-added product of γ-PGA by a glutamate-independent producer of *Bacillus velezensis* GJ11.

**Results:**

FA power could partially substitute the high-cost substrates such as sodium glutamate and citrate sodium for producing γ-PGA. With FA powder in the fermentation medium, the amount of sodium glutamate and citrate sodium used for producing γ-PGA were both decreased around one-third. Moreover, FA powder could completely substitute Mg^2+^, Mn^2+^, Ca^2+^, and Fe^3+^ in the fermentation medium for producing γ-PGA. In the optimized medium with FA powder, the γ-PGA was produced at 42.55 g/L with a productivity of 1.15 g/(L·h), while only 2.87 g/L was produced in the medium without FA powder. Hydrolyzed γ-PGA could trigger induced systemic resistance (ISR), *e.g.,* H_2_O_2_ accumulation and callose deposition, against the pathogen’s infection in plants. Further investigations found that the ISR triggered by γ-PGA hydrolysates was dependent on the ethylene (ET) signaling and nonexpressor of pathogenesis-related proteins 1 (NPR1).

**Conclusions:**

To our knowledge, this is the first report to use the industry waste, FA powder, as a sustainable substrate for microbial synthesis of γ-PGA. This bioprocess can not only develop a new way to use FA powder as a cheap feedstock for producing γ-PGA, but also help to reduce pollution from the wastewater of yeast molasses fermentation.

## Background

Due to the petroleum crisis, renewable sources, such as bioethanol, have been explored and used as alternative energy in recent years [1]. Molasses is one of the main raw materials for producing bioethanol through yeast fermentation [2–4], because it contains many easily fermentable sugars (e.g., primarily sucrose, glucose, and fructose), and is abundant, low cost, and highly available for the fermentation industry [5–7]. Thereby, molasses is widely used for microbial conversion of different value-added products, such as bioethanol, baker’s yeast, amino acids, butanol, organic acids, single-cell proteins, etc. [8–11]. However, the fermentation also causes a severe waste disposal problem worldwide [12], because most wastewater is discharged into the environment without appropriate pre-treatment, aggravating the dire water pollution situations [1, 13, 14].

In China, the wastewater produced during molasses fermentation can be processed into a biological product, fulvic acid (FA) powder, by dry-spraying. This technology can produce approximately 200 thousands of tons of FA powder every year. Thereby, processing wastewater into FA powder is favorable for reducing the pollution from molasses fermentation. Moreover, the FA powder is rich in FA (> 45%) as well as other nutrients, such as N (> 3%), P (> 0.5%), K (> 12%), and amino acids (> 6%), so it can be used as a fertilizer for crops via promoting plant growth and microorganism activity, controlling plant diseases, and protecting plants against abiotic stresses [15–19]. However, excessive application of FA powder can lead to soil acidification, because it contains a lot of organic acids. On the other hand, FA powder is soluble, and contains many nutrients, such as N, P, K, carbon sources, and amino acids, which makes it a kind of cheap raw material (~ 1500 ¥ per ton) for producing value-added products. By fermentation, the organic acids in FA power can be consumed by microorganisms as carbon sources except for FA, which contains a high amount of oxygen-rich and carbon-poor functional groups, so it is hard to be used by microbes [18]. Thereby, the fermented FA powder will be an ideal fertilizer with a minimized side effect on soil acidification. Furthermore, the FA powder can be bioconverted into some value-added products by fermentation that is favorable for encouraging people to convert the wastewater produced during the molasses fermentation into FA powder instead of polluting the environment.

*Bacillus* is ubiquitously distributed in the natural environment. Many *Bacillus* species, such as *B. subtilis*, *B. licheniformis*, *B. amyloliquefaciens*, and *B. velezensis,* are generally used as hosts for producing fermentation products including poly-γ-glutamic acid (γ-PGA). γ-PGA is a natural anionic polymer of d/l-glutamic acids linked together via amide bonds between the α-amino group and the γ-carboxylic acid group, resulting in numerous properties, such as holding water, biodegradability, and non-toxicity [20, 21]. Thereby, it has a wide application in food, medicine, cosmetic, and agriculture nowadays [22]. In agriculture, γ-PGA is a new environmental-friendly fertilizer synergist for improving plant in uptake of N, P, and K [23]. However, its application in agriculture is hindered due to the low yield and high cost of the production when compared with the conventional materials, because the production of γ-PGA via fermentation generally needs glutamate and other high-cost components as substrates. Thereby, some low-cost feedstocks (e.g., FA powder) are urgently needed to overcome the economical and sustainable obstacles to biotechnologically produce γ-PGA.

There are two types of *Bacillus* strains that can produce γ-PGA: one requires an external supply of glutamate and the other does not [21]. We previously isolated a strain of *B. velezensis* GJ11 from soil with an excellent activity to trigger the induced systemic resistance (ISR) in plants [24]. Interestingly, this strain could also produce γ-PGA in a glutamate-independent manner. The aim of this study was to use GJ11 for microbial conversion of FA powder into γ-PGA with low cost, and investigate whether γ-PGA could be used as an activator to trigger ISR in plants [25]. We found that FA powder could reduce the cost of γ-PGA production around one-third, and the γ-PGA produced by GJ11 was able to trigger ISR against pathogens infection via the ethylene (ET) signaling and nonexpressor of pathogenesis-related proteins 1 (NPR1) in plants.

## Results

### GJ11 producing γ-PGA by a glutamate-independent manner

**Fig. 1:**
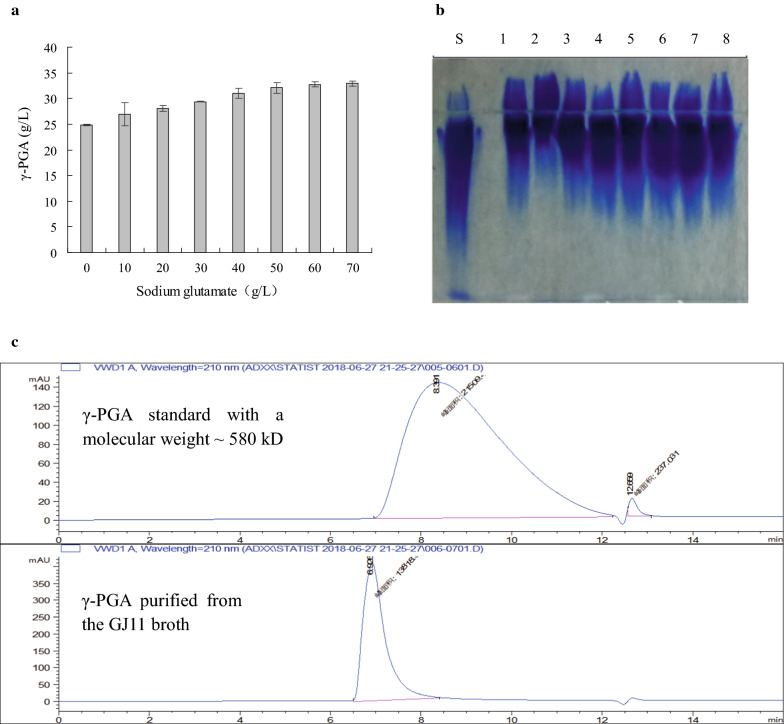
γ-PGA produced by GJ11. **a** Effect of sodium glutamate on γ-PGA production. **b** SDS-PAGE analysis of γ-PGA produced by GJ11 in the medium with sodium glutamate. Lane S: Commercial γ-PGA standard; Lane 1—8: γ-PGA produced by GJ11 in the medium with 70, 60, 50, 40, 30, 20, 10, and 0 g/L of sodium glutamate, respectively. **c** Gel permeation chromatography analysis of the molecular weights of γ-PGA produced by GJ11

Without addition with sodium glutamate in the medium, GJ11 produced ~ 25.0 g/L of γ-PGA in the broth. Addition with sodium glutamate could increase the production of γ-PGA. When the concentration of sodium glutamate was increased, the γ-PGA production was also increased in the broth. After the sodium glutamate concentration was increased more than 40.0 g/L, the γ-PGA production was slightly increased with the increase of sodium glutamate concentration (Fig. 1a). This was consistent with the result of SDS-PAGE (Fig. 1b). Thereby, GJ11 is a glutamate-independent γ-PGA producer, and an addition of glutamate can further improve its γ-PGA production. We further used gel permeation chromatography to determine the molecular weight of γ-PGA produced by GJ11. The average retention time of standard γ-PGA was about 8.39 min, while the average retention time of γ-PGA produced by GJ11 was about 6.92 min (Fig. 1c). It is known that the molecule with a higher molecular weight has a shorter retention time for the gel permeation chromatography. Thereby, the molecular weight of γ-PGA produced by GJ11 is higher than the γ-PGA standard with a molecular weight of 580 kD.

### Compatibility of GJ11 to FA powder

FA powder was dissolved in water as a medium for culturing GJ11. The results showed that the biomass of GJ11 was gradually increased with the increase of FA powder (< 40 g/L), indicating that FA powder could provide nutrients for the bacterial growth when it was used at a concentration less than 40 g/L. After the FA powder concentration was more than 40 g/L, the biomass of GJ11 was decreased with the increase of FA powder added in the medium (Additional file 1: Fig. S1a). Further studies showed that, with the increase of FA powder concentration, the pH value of medium was decreased due to the presence of organic acids in the power (Additional file 1: Fig. S1b). The low pH value suppressed the bacterial growth. Thereby, we adjusted the pH value of medium to 7.0 before sterilization, and found it decreased to ~ 6.5 after sterilization (Additional file 1: Fig. S1c).

The original fermentation medium with FA powder was adjust to pH 7.0 for culturing GJ11. As shown in Additional file 1: Fig. S1d, the biomass of culture reached the maximum when FA powder was used at a concentration of 40 g/L. When the concentration of FA powder was more than 40 g/L, the biomass decreased dramatically with the increase of FA powder added in the fermentation medium. Differently, an addition of FA power could not increase the γ-PGA production. The γ-PGA production reached the highest value, ~ 42 g/L, when no FA powder was added in the fermentation medium. Thereby, addition with FA power in the original fermentation medium could not increase but reduce γ-PGA production in the broth.

### Effects of carbon and nitrogen sources on γ-PGA production

**Fig. 2:**
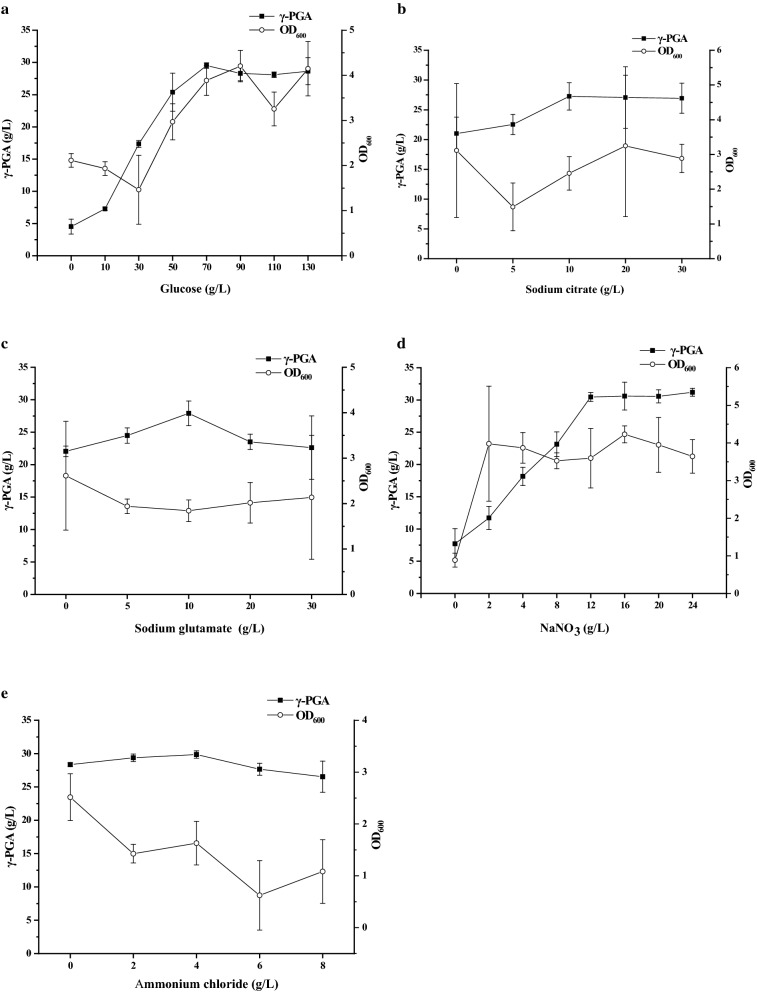
Effects of carbon and nitrogen sources on biomass and γ-PGA production. **a** Glucose; **b** citrate sodium; **c** sodium glutamate; **d** NaNO_3_; **e** NHCl_4_

Addition with FA powder in the original fermentation medium was unfavorable for GJ11 to produce γ-PGA; thereby, we further detected whether FA powder (40 g/L) could substitute some nutrients for reducing the cost of γ-PGA production. Glucose is known as the most efficient carbon source for producing γ-PGA currently. Thus, we studied whether FA powder could substitute glucose in the fermentation medium. As shown in Fig. 2a, the biomass and γ-PGA production were both decreased in the broth when no glucose was added in the medium. Conversely, addition with glucose could increase both of biomass and γ-PGA production in the broth. Under a glucose concentration of 70 g/L, the biomass and γ-PGA production were both increased with the increase of glucose concentration in medium. When the glucose concentration was more than 70 g/L, the biomass and γ-PGA production was no longer increased with the increase of glucose in medium. Besides glucose, citrate acid is also regarded as a common carbon source for γ-PGA production. As shown in Fig. 2b, the γ-PGA production, rather than the biomass, was increased with the increase of citrate sodium concentration in medium. This was probably due to the reason that citrate acid was mainly contributed to biosynthesis of glutamate, a monomer for biosynthesizing γ-PGA, rather than the bacterial growth. When the concentration of citrate sodium was more than 10 g/L, the production of γ-PGA was no longer increased with the increase of citrate sodium added in the medium.

Although GJ11 is a glutamate-independent γ-PGA producer, addition with glutamate could improve its γ-PGA production. As shown in Fig. 2c, the biomass was not significantly influenced by addition with sodium glutamate, but the γ-PGA production was obviously increased by addition with sodium glutamate in the medium. The highest γ-PGA production was obtained when sodium glutamate was added at a final concentration of 10 g/L, and higher concentrations of sodium glutamate could not improve but reduce γ-PGA production in the broth.

The glutamate-independent producer can use inorganic nitrogen sources for biosynthesizing γ-PGA [21]. As shown in Fig. 2d, an addition of NaNO_3_ could improve both biomass and γ-PGA production in the broth. When the concentration of NaNO_3_ was more than 2 g/L, the biomass was no longer increased with the increase of NaNO_3_, but the γ-PGA production was still increased until the concentration of NaNO_3_ reached to 12 g/L. However, another inorganic nitrogen source, NH_4_Cl, could neither improve γ-PGA production nor increase biomass in the broth (Fig. 2e). Moreover, the bacterial growth was inhibited by NH_4_Cl when it was added in the medium at a concentration more than 2 g/L. Similarly, the γ-PGA production was reduced when NH_4_Cl was added in the medium at a concentration more than 4 g/L.

### Effects of inorganic salts on γ-PGA production

**Fig. 3:**
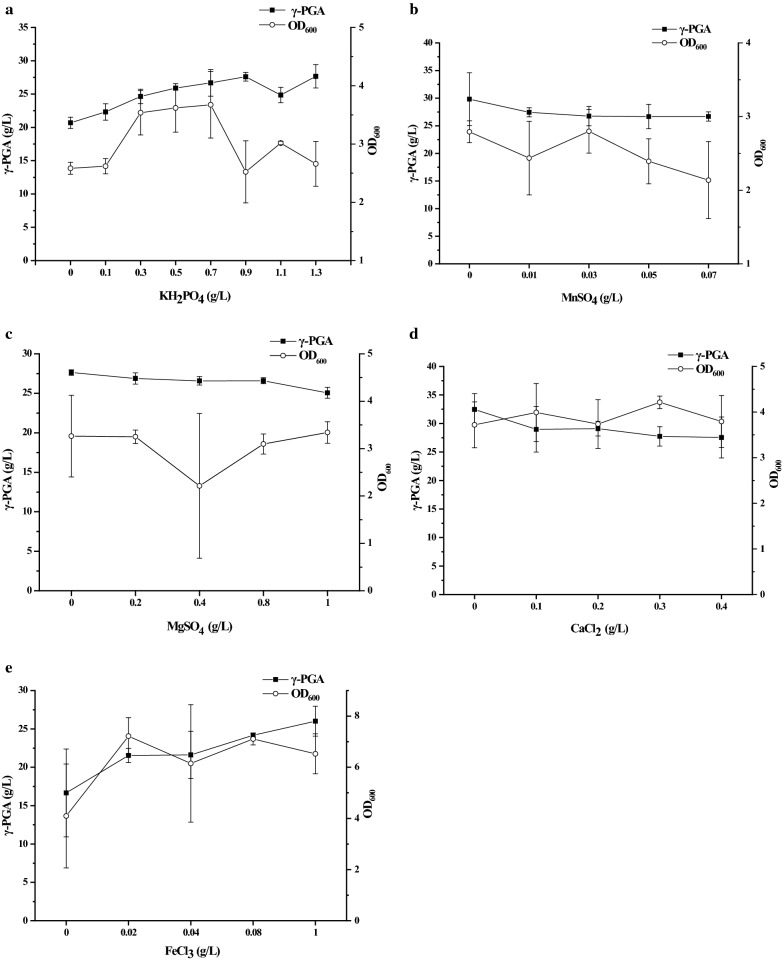
Effects of inorganic salts on biomass and γ-PGA production in the medium with FA power. **a** KH_2_PO_4_; **b** MnSO_4_; **c** MgSO_4_; **d** CaCl_2_; **e** FeCl_3_

Inorganic salts have been reported to be important for γ-PGA production [26, 27]. As shown in Fig. 3a, KH_2_PO_4_ could improve both biomass and γ-PGA production in the broth. However, when the concentration of KH_2_PO_4_ was more than 0.3 g/L, the biomass was no longer increased with the increase of KH_2_PO_4_ added in the medium. Moreover, the biomass was reduced when the concentration of KH_2_PO_4_ was more than 0.7 g/L. Similarly, the excessive KH_2_PO_4_ (> 0.9 g/L) was also unfavorable for GJ11 to produce γ-PGA.

It has been reported that Mn^2+^ can improve cell growth, prolong cell viability, and assist the utilization of different carbon sources [26]. As shown in Fig. 3b, an addition of MnSO_4_ could not significantly improve γ-PGA production, indicating that Mn^2+^ in the FA powder was enough for GJ11 to produce γ-PGA. Moreover, with the increase of MnSO_4_ added in the medium, the γ-PGA production was gradually decreased in broth.

Mg^2+^ is necessary for the activity of PgsBCA, the enzyme complex for biosynthesizing γ-PGA in *Bacillus* [27]. Our results showed that an addition of MgSO_4_ could not improve biomass and γ-PGA production in the broth. When the concentration of MgSO_4_ increased, the γ-PGA production was gradually reduced in broth (Fig. 3c). Thereby, an addition of Mg^2+^ was not necessary for producing γ-PGA in the medium with FA powder.

We further investigated whether an addition of Ca^2+^ could improve γ-PGA production in the broth. It was found that an addition of CaCl_2_ could not significantly improve biomass and γ-PGA production in the medium with FA powder. Moreover, the excessive Ca^2+^ even inhibited the γ-PGA production in broth (Fig. 3d). The results indicated that Ca^2+^ was already enough for producing γ-PGA in the medium with FA powder.

As shown in Fig. 3e, an addition of FeCl_3_ in the medium with FA powder was favorable for improving biomass rather than γ-PGA production in the broth. However, the excessive Fe^3+^ (> 0.02 g/L) could not further improve biomass in the broth.

### Orthogonal test for optimizing fermentation medium

On the basis of above results, we further optimized the fermentation medium formula with orthogonal test. Glucose, citrate sodium, sodium glutamate, NaNO_3_, KH_2_PO_4_, and FA powder were selected for further optimization according to the orthogonal experiments ([L18(3^7^)]. As shown in Table 1, glucose, NaNO_3_, sodium glutamate, and citrate sodium improved the γ-PGA production, while FA powder was negative for the γ-PGA production. According to R value obtained from the orthogonal tests, we found that γ-PGA production was successively affected by sodium glutamate, NaNO_3_, citrate sodium, FA powder, glucose, and KH_2_PO_4_. The optimal combination of medium was A3B3C2D1E3F3, corresponding to 80 g/L glucose, 20 g/L NaNO_3_, 0.7 g/L KH_2_PO_4_, 20 g/L FA powder, 20 g/L sodium glutamate, and 20 g/L citrate sodium, which resulted in the highest γ-PGA production of 35.54 g/L. On the other hand, glucose and FA powder were favorable, while NaNO_3_, sodium glutamate, citrate sodium, and KH_2_PO_4_ were unfavorable, for the bacterial growth. On the basis of R value, we found that the biomass was successively affected by glucose, NaNO_3_, citrate sodium, FA powder, sodium glutamate, and KH_2_PO_4_. The bacterial growth showed a negative correlation with the γ-PGA production of GJ11. Here, we mainly focused on the γ-PGA production, so the optimized medium formula was selected as 80 g/L glucose, 20 g/L NaNO_3_, 0.5 g/L KH_2_PO_4_, 20 g/L FA powder, 20 g/L sodium glutamate, and 20 g/L citrate sodium. Compared to the original fermentation medium, the cost of sodium glutamate and citrate sodium were both decreased around one-third due to an addition of FA powder in the medium. Moreover, FA powder could be a substitute for NH_4_Cl, MgSO_4_, MnSO_4_, CaCl_2_, and FeCl_3_ in the original fermentation medium.

**Table 1:** Results of orthogonal experiment

Treatment	A	B	C	D	E	F	γ-PGA (g/L)	OD_600_
1	1	1	1	1	1	1	15.39	4.91
2	1	2	2	2	2	2	22.08	3.04
3	1	3	3	3	3	3	27.14	2.15
4	2	1	1	2	2	3	22.51	5.27
5	2	2	2	3	3	1	26.83	5.52
6	2	3	3	1	1	2	25.53	3.61
7	3	1	2	1	3	2	29.86	6.88
8	3	2	3	2	1	3	26.69	7.03
9	3	3	1	3	2	1	22.78	7.38
10	1	1	3	3	2	2	17.69	4.98
11	1	2	1	1	3	3	33.06	3.00
12	1	3	2	2	1	1	21.68	4.42
13	2	1	2	3	1	3	19.09	5.20
14	2	2	3	1	2	1	20.87	4.12
15	2	3	1	2	3	2	29.21	4.45
16	3	1	3	2	3	1	24.31	7.01
17	3	2	1	3	1	2	22.23	7.20
18	3	3	2	1	2	3	35.54	4.43
K1	138.44	128.86	145.19	160.25	130.62	131.87		
K2	144.04	151.76	155.08	146.48	141.48	146.60		
K3	161.42	161.88	142.24	135.77	170.41	164.04		
k1	23.07	21.48	24.20	26.71	21.77	21.98		
k2	24.01	25.29	25.85	24.41	23.58	24.43		
k3	26.90	26.98	23.71	22.63	28.40	27.34		
R	3.83	5.50	2.14	4.08	6.63	5.36		

### Optimization of fermentation conditions for γ-PGA production

**Fig. 4:**
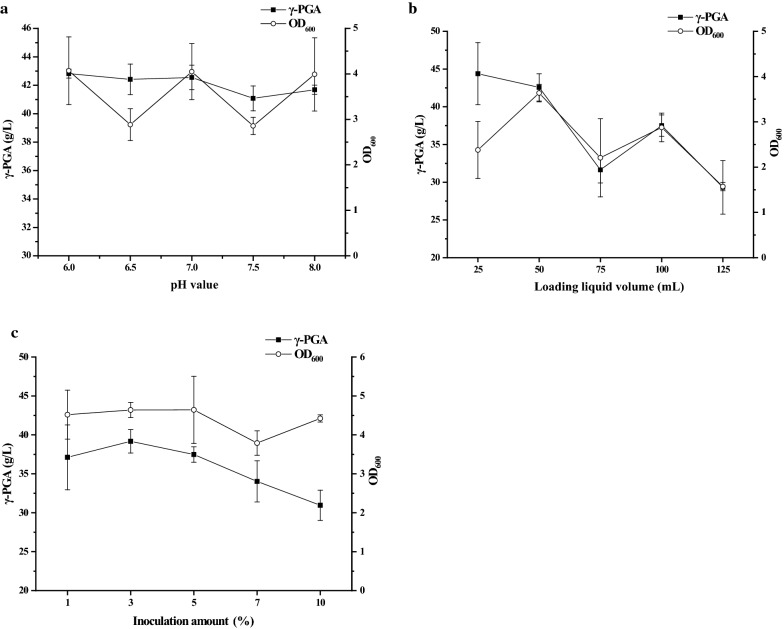
Optimization of fermentation condition for production of γ-PGA.** a** Effects of pH on cell biomass and γ-PGA production. **b** Effects of loading liquid volume on cell growth and γ-PGA production. **c** Effects of inoculation amount on cell growth and γ-PGA production

With the optimized fermentation medium formula, we further detected the effect of pH on γ-PGA production. As shown in Fig. 4a, the increase of medium pH value led to a gradually decreased γ-PGA production in the broth. The biomass was changed with the change of medium pH value, and at pH 7.0, the biomass achieved the highest value. The biomass was decreased at pH 6.5, increased at pH 7.0, then decreased again at pH 7.5, and increased again at pH 8.0. This might be due to the reason that the FA power contains different organic acids. The change of pH value might influence the dissociation and solubility of organic acids in the medium. Different organic acids have different dissociation and solubility at different pH values. Thereby, the availability of organic acids to be used by the bacterium was possibly changed along with the change of medium pH value. Due to this reason, the biomass was also changed with the change of medium pH value.

We also investigated the effect of liquid volume on γ-PGA production, and found the γ-PGA production was decreased when the liquid volume was increased in the flask (Fig. 4b). The biomass reached the highest value when the liquid volume was 50 mL which was loaded in a 250 mL flask, and the excessive liquid volumes were unfavorable for the bacterial growth. As shown in Fig. 4c, the γ-PGA production and biomass were both influenced by inoculation amount. When the inoculation amount was more than 3%, the γ-PGA production was gradually decreased with the increase of inoculation amount. Similarly, the biomass was also decreased with the increase of inoculation amount (> 5%).

### Verification of optimized fermentation medium formula and conditions for γ-PGA production

**Fig. 5:**
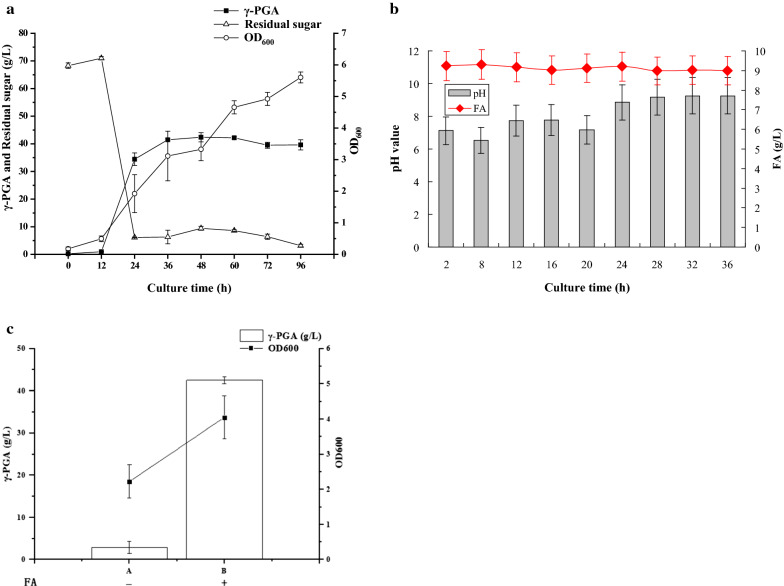
Optimized fermentation medium and condition for γ-PGA production. **a** Time-course of γ-PGA fermentation. **b** Variation of pH value and FA content in the broth. **c** Influence of FA power on biomass and γ-PGA production in the optimized formula

We cultured GJ11 in the optimized fermentation medium and conditions, and found glucose in the medium was not obviously consumed by GJ11 during the first 12 h, corresponding to the result that no γ-PGA was accumulated in this period. During 12—36 h, the biomass was dramatically increased, corresponding to a rapid decrease of residual glucose in the broth. Consistently, γ-PGA was rapidly biosynthesized in this period, with a maximum production of 41.47 g/L and a high productivity of 1.15 g/(L·h). During 36—48 h, the biomass, residual glucose, and γ-PGA production had no obvious change. During 48—96 h, the biomass was increased again, accompanied by a decrease of residual glucose in the broth. In this period, γ-PGA production was gradually decreased (Fig. 5a), suggesting that some γ-PGA was consumed as carbon and nitrogen source for the bacterial growth. FA in the medium could not be used by GJ11, which was consistent with the previous report [18]. However, other organic acids could be gradually consumed by GJ11, accompanied by a gradually increased pH value of broth (Fig. 5b).

We further verified the influence of FA powder on biomass and γ-PGA production in the optimized formula. It was found that 42.55 g/L of γ-PGA was produced with the optimized fermentation medium containing 20 g/L FA powder, while only 2.87 g/L γ-PGA was produced with the medium without FA powder (Fig. 5c). These results indicated that FA powder was very important for the γ-PGA production in our optimized formula, because it contains some necessary nutrients for producing this polymer.

### Hypersensitive response (HR) and induced systemic resistance (ISR) triggered by γ-PGA and its hydrolysates

**Fig. 6:**
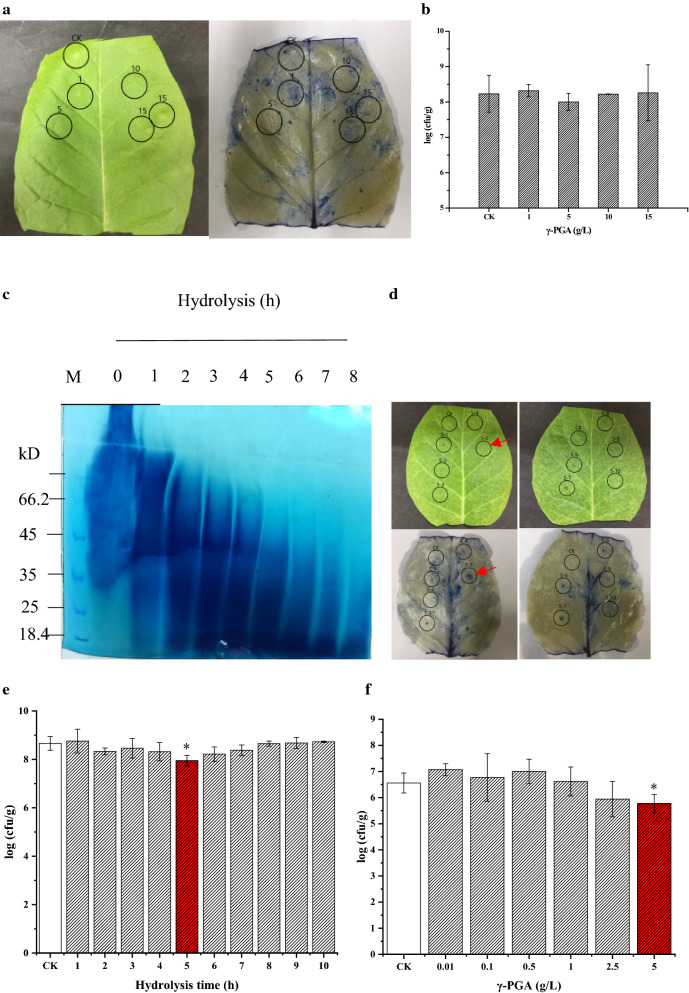
Effects of γ-PGA and its hydrolysates on triggering HR and ISR against *Pst* DC3000 infection in tobacco plants. **a** Effect of γ-PGA on triggering HR in tobacco leaves; **b** Effect of γ-PGA on triggering ISR against *Pst* DC3000 infection in tobacco plants. **c** SDS-PAGE analysis of γ-PGA hydrolysates. The pH of γ-PGA solution (5 g/L) was adjusted to 2.0, then hydrolyzed at 80 °C for 0, 1, 2, 3, 4, 5, 6, 7, and 8 h, respectively. **d** Effect of γ-PGA hydrolysates on triggering HR in tobacco plants. 5–1—5–10: 5 g/L γ-PGA hydrolysates produced by digestion at pH 2.0 and 80 °C for 1, 2, 3, 4, 5, 6, 7, 8, 9, and 10 h, respectively. **e** ISR triggered by 5 g/L γ-PGA hydrolysates produced by digestion at pH 2.0 and 80 °C for 1, 2, 3, 4, 5, 6, 7, 8, 9, and 10 h, respectively, to protect plants from *Pst* DC3000 infection. **f** Effect of γ-PGA hydrolysates (digestion at pH 2.0 and 80 °C for 5 h) at different concentrations on triggering ISR against *Pst* DC3000 infection. γ-PGA: the γ-PGA hydrolysates. *indicates significant difference from the control (CK, treated with water)

Lei et al. have reported that γ-PGA can protect plants against abiotic stress, such as high and low temperature [28]. In this study, we used γ-PGA purified from the broth of GJ11 to treat plants, and then invested whether HR and ISR could be triggered in the leaves. The results showed that γ-PGA could not induce neither HR nor ISR to protect plants form the pathogen (*Pst* DC3000) infection (Fig. 6a, b). We further digested γ-PGA into the hydrolysates with smaller molecular weights, and detected whether the γ-PGA hydrolysates could induce the HR and ISR in plants. It was found that, as the hydrolysis time increased, more and more γ-PGA was digested into the hydrolysates with smaller molecular weights (Fig. 6c). After hydrolysis, the pH value of solution was adjusted to 7.0, and then used for injecting the tobacco leaves by a syringe without needles. As shown in Fig. 6d, the γ-PGA hydrolysates, which were produced by digestion of γ-PGA at pH 2.0 and 80 °C for 5 h, could trigger HR in the leaves significantly. Consistently, the plants with ISR triggered by irrigating roots with the γ-PGA hydrolysates showed a significant resistance against the pathogen (*Pst* DC3000) infection (Fig. 6e). Further studies found that 5 g/L γ-PGA hydrolysates were more effective for inducing the plant resistance against the *Pst* DC3000 infection (Fig. 6f).

### *H*_*2*_*O*_*2*_* accumulation and callose deposition induced by γ-PGA hydrolysates*

**Fig. 7:**
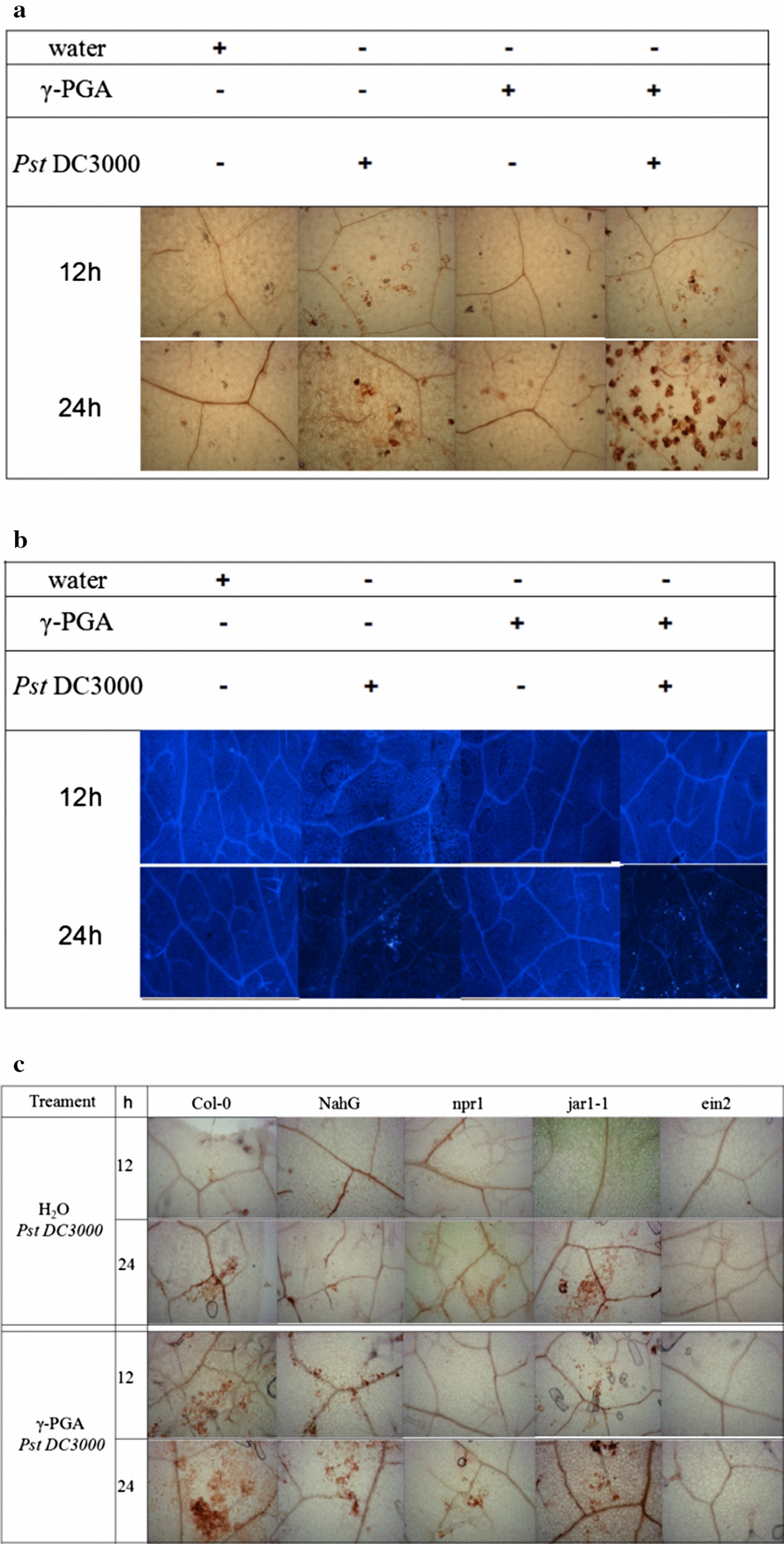
H_2_O_2_ accumulation and callose deposition in the leaves treated with γ-PGA hydrolysates. **a** H_2_O_2_ accumulation in the leaves of wild-type line of *Arabidopsis* pre-treated with γ-PGA hydrolysates following with infection by *Pst* DC3000. **b** Callose deposition in the leaves of wild-type line of *Arabidopsis* pre-treated with γ-PGA hydrolysates following with infection by *Pst* DC3000. **c** H_2_O_2_ accumulation in the leaves of mutant lines of *Arabidopsis* pre-treated with γ-PGA hydrolysates following with infection by *Pst* DC3000. **d** Callose deposition in the leaves of mutant lines of *Arabidopsis* pre-treated with γ-PGA hydrolysates following with infection by *Pst* DC3000. **e** Pathogen amounts recovered from the leaves of mutant lines of *Arabidopsis* pre-treated with γ-PGA hydrolysates following with infection by *Pst* DC3000. *indicates significant difference from the control (CK, pre-treated with water). γ-PGA: the γ-PGA hydrolysates

Many activators produced by beneficial microorganisms can trigger ISR to protect plants from pathogens infection by eliciting the defense-related responses, such as reactive oxygen species (ROS, e.g., H_2_O_2_) accumulation and callose deposition [24]. To know whether the γ-PGA hydrolysates could induce the defense-related responses, the roots of *Arabidopsis thaliana* were treated with the γ-PGA hydrolysates and then infected by *Pst* DC3000. The results showed that treatment with the γ-PGA hydrolysates alone could not induce significant H_2_O_2_ accumulation (Fig. 7a) and callose deposition (Fig. 7b) in the leaves. However, inoculation with pathogen alone could elicit H_2_O_2_ accumulation and callose deposition in the leaves. Further investigation found that pre-treatment with the γ-PGA hydrolysates could elicit a mild but effective plant immunity rapidly responding to the *Pst* DC3000 infection, accompanied by an obvious H_2_O_2_ accumulation (Fig. 7a) and callose deposition (Fig. 7b) in the leaves.

We used different defense-compromised lines of *Arabidopsis*, including NahG (salicylic acid (SA)—degrading transgenic line), jar1-1 (jasmonic acid (JA)—insensitive line), ein2 (ethylene (ET)—insensitive line), and npr1 (nonexpressor of PR proteins), to detect possible signaling induced by the γ-PGA hydrolysates. Compared to the control (water), after inoculation with *Pst* DC3000 for 12 h, the lines, including Col-0, NahG, and jar1-1, were all observed with obvious H_2_O_2_ accumulation (Fig. 7c) and callose deposition (Fig. 7d) in the leaves, while the lines, such as ein2 and npr1, were not. After inoculation with *Pst* DC3000 for 24 h, the enhanced H_2_O_2_ accumulation (Fig. 7c) and callose deposition (Fig. 7d) was observed in the lines Col-0, NahG, and jar1-1, rather than in the lines ein2 and npr1, pre-treated with the γ-PGA hydrolysates. These results suggested that the ISR induced by γ-PGA hydrolysates is dependent on NPR1, and the ET signaling, rather than the SA and JA signaling in plants.

We further recovered the pathogen in different lines, and found that pre-treatment with the γ-PGA hydrolysates could significantly reduce the amount of *Pst* DC3000 in Col-0, NahG, and jar1-1 when compared to that in the control (CK) (Fig. 7e). However, the pathogen amounts recovered from ein2 and npr1 was similar between the group pre-treated with γ-PGA hydrolysates and the control (CK). The results further verified that the ISR induced by γ-PGA hydrolysates is dependent on the ET signaling and NPR1 in plants.

## Discussion

Previously, we isolated a strain *B. velezensis* GJ11, which could produce acetoin to trigger ISR in plants [24]. Here, we found it could also produce γ-PGA using glucose and ammonium chloride as substrates in a glutamate-independent manner. However, most of these producers have low yield of γ-PGA. Thus, people began to use glutamate to enhance the yield of this polymer, resulting in a significant increase of the cost. To produce γ-PGA at a low cost, we tested whether FA powder, which is produced from the wastewater of molasses fermentation in the yeast fermentation industry [29], could substitute some nutrients in the original fermentation medium formula. We first determined whether GJ11 was compatible to FA powder. Due to the low pH of FA powder medium, increasing the concentration of FA powder resulted in a gradual decrease of medium pH value, which was unfavorable for the bacterial growth. However, the decrease could be reversed by increasing the original pH value of the medium with NaOH. pH is also an important environmental factor for γ-PGA fermentation. It has been reported that pH 6.5 can support a high γ-PGA production, whereas pH 7.0 is favorable for the cell growth [26]. Thereby, to enhance γ-PGA production, the broth pH should be maintained at 7.0 for the first 24 h to obtain a maximum biomass, and then shifted to 6.5 to maximize γ-PGA production.

Although FA powder could promote the growth of GJ11 at certain concentrations, it was unfavorable for the cells to produce γ-PGA. Thereby, we further optimized the medium formula with FA powder to improve the γ-PGA production and reduce the production cost. Generally, glucose is a preferred carbon source for producing γ-PGA [21]. In this study, an addition of glucose could effectively improve both biomass and γ-PGA production in the medium with FA powder, indicating that FA powder alone is not enough to provide carbon sources for producing γ-PGA. Thereby, FA powder could not substitute glucose in the original fermentation medium formula. Production of γ-PGA often requires the supplementation of glutamate, resulting in an increase in the overall cost of production [15]. Although GJ11 is a glutamate-independent producer, our study showed that an addition of glutamate could also significantly increase its γ-PGA production. Interestingly, FA powder could partially substitute glutamate in the original fermentation medium formula. Glutamate-independent producer can de novo synthesize glutamate monomer for biosynthesis of γ-PGA via the TCA cycle, so citrate acid is able to enhance γ-PGA production by joining the TCA cycle directly to elevate the intracellular level of α-ketoglutarate [30]. In this way, more glutamate are generated for producing γ-PGA as a substrate [21, 31]. Here, we tested whether FA powder could substitute citric acid in the original fermentation medium formula for producing γ-PGA. According to our results, the FA powder with organic acids could also partially substitute citric acid in the medium for producing γ-PGA.

GJ11 can use inorganic nitrogen sources to synthesize glutamate for γ-PGA production. In this study, we found that FA powder could substitute NH_4_Cl rather than NANO_3_ in the original fermentation medium formula, indicating that FA powder mainly contains NH_4_^+^ rather than nitrate. Mg^2+^ is necessary for γ-PGA production, because it is critical for the activity of PgsBCA that is responsible for biosynthesis of γ-PGA in *Bacillus* [27]. Mn^2+^ is important for the stereochemical and enantiomeric composition of γ-PGA [26]. Ca^2+^ and Fe^3+^ are also needed for high production of γ-PGA. In this study, we found that FA powder could substitute all of these ions listed above. As a result, an addition of 20 g/L FA powder could decrease the cost of glutamate and citrate acid around one-third, and completely substitute NH_4_^+^, Mg^2+^, Mn^2+^, and Fe^3+^ when compared to the original fermentation medium formula. Thereby, the formula of medium with FA powder can greatly reduce the cost for producing γ-PGA via fermentation.

Although *B. subtilis* and *B. licheniformis* are promising native bacteria for commercial production of γ-PGA most frequently [21], our study proved that *B. velezensis* GJ11 is also potential for γ-PGA production. With the optimized fermentation medium and conditions, the γ-PGA production reached a maximum concentration of 42.55 g/L, and a high productivity of 1.15 g/(L·h). In this formula, FA powder is essential, because the medium without FA powder had a much lower production of γ-PGA which was only 2.87 g/L in the broth.

After fermentation, the pH value of broth was significantly increased and FA was slightly consumed by GJ11. Thereby, the broth containing both γ-PGA and FA with a high pH value should be a better and more effective fertilizer than the FA power alone. In agriculture, γ-PGA is also reported with the ability to protect crops from plant diseases (*e.g., Fusarium* root rot) in addition to being used as a fertilizer synergist [32]. We hypothesized that γ-PGA might act as an activator to induce resistance against the pathogen infection in plants. Plant activators that can induce defense response have attracted increasing attentions due to their potentials in controlling plant diseases while reducing the environmental burdens. Their action mechanisms can activate a complex signaling network, including the pathways regulated by salicylic acid (SA), ethylene (ET), jasmonic acid (JA), etc. [19]. In this study, we investigated whether γ-PGA could trigger resistance against the pathogens infection in plants. We found that the γ-PGA with high molecular weights could not effectively trigger the resistance against *Pst* DC3000 infection, but the γ-PGA hydrolysates were effective to induce the defense response (e.g., HR, H_2_O_2_ accumulation and callose deposition) against the pathogen infection. Our results also revealed that the γ-PGA hydrolysates mainly triggered ISR via the ET signaling and NPR1 in plants. Thereby, in addition to being used as a fertilizer synergist, γ-PGA is potential to be used as a new activator to trigger the defense response against plant diseases.

## Conclusions

We used the fermentation industry waste, FA powder, as a sustainable substrate for microbial synthesis of some biotechnological products such as γ-PGA. This technology can not only alleviate the soil acidification induced by directly returning FA powder into soil, but also develop a new application of FA powder as a cheap raw material for producing γ-PGA at a low cost. Moreover, the novel use of FA powder as a raw material for fermentation is favorable for reducing the possible pollution induced by wastewater from the yeast fermentation with molasses. Thereby, the bioprocess of converting FA powder to highly valuable products, such as γ-PGA, is circular economic.

## Materials and methods

### Strains, mediums, and chemicals

The strain used for producing γ-PGA was *B. velezensis* GJ11 [24]. The lines of *Arabidopsis thaliana*, including a wild-type line Col-0, and four defense-compromised mutants which were npr1 (nonexpressor of PR proteins), jar1-1 (jasmonic acid (JA)—insensitive line), ein2 (ethylene (ET)—insensitive line), and NahG (salicylic acid (SA)—degrading transgenic line) [33], were gifts from *Prof*. Yan S, Huazhong Agricultural University, Wuhan, Hubei, China. FA powder was bought from Lesaffre, Guangxi, China. All other chemicals were of analytical grade supplied by Sinopharm Chemical Reagent (China).

The basic fermentation medium (pH 7.4) contained (g/L): glucose 70.0, citrate sodium 30.0, NaNO_3_ 12.0, NH_4_Cl 8.0, K_2_HPO_4_ 0.7, CaCl_2_ 0.3, MgSO_4_·7H_2_O 1.0, MnSO_4_·H_2_O 0.07, and FeCl_3_·6H_2_O 0.08. The original fermentation medium was prepared with the basic fermentation medium and sodium glutamate (30.0 g/L).

### Production of γ-PGA by GJ11

We determined whether GJ11 could produce γ-PGA by a glutamate-independent manner. GJ11 was cultured in LB medium overnight, then inoculated into 50 mL of the basic fermentation medium or the basic fermentation medium with a concentration gradient of sodium glutamate (10, 20, 30, 40, 50, 60 and 70 g/L, respectively) in a 250 mL flask, and incubated at 37 °C and 180 rpm for 36 h. After the incubation, the samples in different broths were collected for determining the γ-PGA produced by GJ11.

### Detecting compatibility of GJ11 to FA powder

GJ11 was cultured in LB medium overnight and then inoculated into 50 mL of the concentration—gradient FA powder medium (1, 5, 10, 20, 40, 60 and 100 g/L, respectively) in a 250 mL flask at a ratio of 3% (v/v), for further culture at 37 °C and 180 rpm for 24 h. After the incubation, the samples in different broths were collected for counting the colony-forming unit (*cfu*) of GJ11.

### Assaying influences of FA powder on γ-PGA production and biomass

FA powder was added into the original fermentation medium at different concentrations (0, 10, 20, 40, 60, and 100 g/L, respectively). The medium pH value was adjusted to 7.0 using 6 M NaOH solution. GJ11 was cultured in LB medium overnight and then transferred into 50 mL of the original fermentation medium with FA powder in a 250 mL flask at a ratio of 3%, for further culture at 37 °C and 180 rpm for 36 h. After the incubation, the samples were collected for determining γ-PGA production and biomass of the broth.

### Optimizing fermentation medium

In the original fermentation medium, the concentration of FA powder was set at 40 g/L, then the impacts of glucose, citrate sodium, sodium glutamate, NaNO_3_, KH_2_PO_4_, NH_4_Cl, MgSO_4_, MnSO_4_, CaCl_2_, and FeCl_3_ on γ-PGA production and biomass were investigated, respectively. In detail, glucose was added into the medium at a final concentration of 0, 10, 30, 50, 70, 90, 110, and 130 g/L, citrate sodium was added at a final concentration of 0, 5, 10, 20, and 30 g/L, and sodium glutamate was added at a final concentration of 0, 5, 10, 20, and 30 g/L respectively. NaNO_3_ was added into the medium at a final concentration of 0, 2, 4, 8, 12, 16, 20, and 24 g/L, and NH_4_Cl was added at a final concentration of 0, 2, 4, 6 and 8 g/L, respectively. The concentration of KH_2_PO_4_ was set at 0, 0.3, 0.5, 0.7, 0.9, 1.1, and 1.3 g/L, respectively. MnSO_4_ was added into medium at a final concentration of 0, 0.01, 0.03, 0.05, and 0.07 g/L, MgSO_4_ was added at a final concentration of 0, 0.2, 0.4, 0.8, and 1 g/L, CaCl_2_ was added at a final concentration of 0, 0.1, 0.2, 0.3, and 0.4 g/L, and FeCl_3_ was added at a final concentration of 0, 0.02, 0.04, 0.08, and 0.1 g/L, respectively. All other factors were held constantly.

On the basis of above optimization, the orthogonal test was set up for further optimizing the fermentation medium. Glucose, citrate sodium, sodium glutamate, NaNO_3_, KH_2_PO_4_, and FA powder were selected for the orthogonal experiment design [L18(3^7^)], as listed in Table 2.

**Table 2:** Factorial level

Factorial level	A—glucose g/L	B—NaNO_3_g/L	C—KH_2_ PO_4_g/L	D—FA power g/L	E—l-glutamate g/L	F—citrate sodium g/L
1	60	12	0.5	20	5	5
2	70	16	0.7	30	10	10
3	80	20	0.9	40	20	20

### Optimization of culture conditions

On the basis of the orthogonal experiment, we further studied the effect of culture conditions on γ-PGA production. To study the impact of pH on γ-PGA production, the initial pH value of medium was set at 6.0, 6.5, 7.0, 7.5, and 8.0, respectively. To study the impact of ventilation on γ-PGA production, several 250 mL flasks containing 25, 50, 75, 100, and 125 mL medium, respectively, were prepared for culturing GJ11. The amounts of inoculation were set at 1%, 3%, 5%, 7%, and 10% (v/v), respectively, for culturing GJ11 to produce γ-PGA. All other factors were held constantly.

### Detecting γ-PGA production and molecular weight, biomass, residual glucose, pH, and FA of broth

The biomass of GJ11 was assayed by detecting the OD_600_ value of broth. The γ-PGA production was determined by the cetyltrimethylammonium bromide (CTAB) turbidimetry method, and the SDS-PAGE analysis with methylene blue staining [30, 32, 34]. Residual glucose in the broth was determined using SBA-40D Bio-analyzer (Shandong Academy of Sciences, China) [35]. The pH value of broth was detected using a pH meter. The content of fulvic acid was determined by the KMnO_4_ oxidation method. After dissolution, the content of fulvic acid was valued by titration with ferrous ammonium sulfate and N-phenyl anthranilic acid, which could indicate the end point [17, 36]. The γ-PGA molecular weights were measured using gel permeation chromatography with a RI-10 refractive-index detector and a SuperposeTM 6 column (Shimadzu Corp, Japan) [37].

### Analysis of hypersensitive reaction (HR) induced by γ-PGA and its hydrolysates

γ-PGA was recovered from the GJ11 broth [20]. 20 μL of γ-PGA in gradient concentration (1, 5, 10, and 15 g/L, respectively) was used for injecting the tobacco leaves with a 1 mL syringe without needles. After 24 h, the hypersensitive response (HR) was detected via trypan blue staining [24]. γ-PGA was also used for irrigating tobacco seedlings to detect the induced systemic response (ISR) in plants. In detail, the tobacco seedlings planted in pots (one plant per pot, and ten pots per group) were irrigated with γ-PGA solution (1, 5, 10, and 15 g/L, respectively) at 5 ml per seedling. After 3 days, the pathogen of *Pseudomonas syringae* pv. *tomato* DC3000 (termed as *Pst* DC3000, 1 × 10^8^ cfu mL^−1^) was used for infecting tobacco plants by spraying the leaves evenly. The leaves were collected 3 days after the infection, and then sterilized and homogenized for spreading plates. After incubation at 37 °C for 48 h, the bacterial colonies were counted for calculating the *Pst* DC3000 content per gram of fresh leaf (*cfu* g^−1^) [24].

The γ-PGA solution (5 g/L) was adjusted to pH 2.0, and then incubated at 80 °C for 0, 1, 2, 3, 4, 5, 6, 7, and 8 h, respectively. After digestion, the pH value of solution was adjusted to 7.0 with 6 M NaOH, and then, the samples were collected for analysis of the hydrolysates using SDS-PAGE [32]. Thereafter, the γ-PGA hydrolysates were detected for the activity to trigger HR, and the ISR against *Pst* DC3000 infection as above.

### Detecting cellular defensive responses induced by γ-PGA hydrolysates

Different lines of *A. thaliana* seedlings (6-week-old, 10 seedlings per group), including Col-0, NahG, npr1, jar1-1, and ein2, were irrigated with 5 mL of the γ-PGA hydrolysates (5 g/L of γ-PGA was hydrolyzed at pH 2.0 and 80 °C for 5 h), and then infected with *Pst* DC3000 by spraying the leaves as above. After infection for 12 and 24 h, the leaves were collected and stained with DAB to detect the accumulation of H_2_O_2_, a kind of reactive oxygen species (ROS), in plants [24]. The callose deposition in leaves was also detected by our previous methods [24]. Briefly, the leaves of *A. thaliana* were fixed with 1% (v/v) glutaraldehyde solution overnight, treated with ethanol at 90 C for 15 min, and stained with 0.1% (w/v) aniline blue for 12 h, and then observed by fluorescence microscope after being extensively washed by 50% (v/v) ethanol solution.

### Statistical analysis

All experiments were repeated in triplicates. The data between two groups were compared using Student's *t* test.

## Supplementary information

**Additional file 1: Figure S1.** Compatibility of GJ11 to FA power. a Influence of FA power on the growth of GJ11. b Influence of sterilization on pH of the medium with different concentrations of FA power with a natural pH. c Influence of sterilization on pH of the medium with different concentrations of FA power with an original pH of 7.0. d Effect of FA power on cell biomass and γ-PGA production.

## Data Availability

All data generated during this study are included in this published article and its additional file.

## Abbreviations

FA: Fulvic acid
γ-PGA: Poly-γ-glutamic acid
SDS-PAGE: Dodecyl sulfate sodium salt (SDS)-polyacrylamide gel electrophoresis
HR: Hypersensitive response
ISR: Induced systemic resistance
ET: Ethylene
SA: Salicylic acid
JA: Jasmonic acid
CK: Control

## Supplementary information

**Supplementary information** accompanies this paper at 10.1186/s13068-020-01818-5 .
