# Supplementary material for: Microbial synthesis of poly-γ-glutamic acid (γ-PGA) with fulvic acid powder, the waste from yeast molasses fermentation
Source: Biotechnol Biofuels. 2020 Oct 28;13:180. doi: 10.1186/s13068-020-01818-5 (PMC7594462; doi:10.1186/s13068-020-01818-5)
Supplement: Supplementary file 1 — Additional file 1: Figure S1. Compatibility of GJ11 to FA power. a Influence of FA power on the growth of GJ11. b Influence of sterilization on pH of the medium with different concentrations of FA power with a natural pH. c Influence of sterilization on pH of the medium with different concentrations of FA power with an original pH of 7.0. d Effect of FA power on cell biomass and γ-PGA production. [file 13068_2020_1818_MOESM1_ESM.docx]

**Fig. S1**

a b

c d

**Fig. S1 Compatibility of GJ11 to FA power. a:** Influence of FA power on the growth of GJ11. **b:** Influence of sterilization on pH of the medium with different concentrations of FA power with a natural pH. **c:** Influence of sterilization on pH of the medium with different concentrations of FA power with an original pH of 7.0. **d:** Effect of FA power on cell biomass and γ-PGA production.
